# Growth phase-specific induction of a viable but nonculturable (VBNC) state in *Listeria innocua* in response to a green food sanitizer

**DOI:** 10.1128/spectrum.03875-25

**Published:** 2026-03-17

**Authors:** Esther W. Mwangi, Moshe Shemesh, Victor Rodov

**Affiliations:** 1 Agricultural Research Organization, Institute of Postharvest and Food Sciences, The Volcani Institute 196691 , Rishon LeZion, Israel; 2 The Robert H. Smith Faculty of Agriculture, Food and Environment, The Hebrew University of Jerusalem, Institute of Biochemistry, Food Science and Nutrition 26742, Rehovot, Israel; University of Mississippi, University, Mississippi, USA

**Keywords:** gram-positive bacteria, green antimicrobials, flow cytometry, membrane integrity, membrane potential, electron transport, viable but nonculturable (VBNC), growth phase

## Abstract

Sustainable food sanitation and preservation technologies are highly needed to meet the increasing demand for healthy and safe food. In this work, we investigated the effects of a recently developed antimicrobial triple formulation (TF) comprised of a natural polyphenol gallic acid amended with the generally recognized as safe materials hydrogen peroxide and DL-lactic acid, on a gram-positive bacterium *Listeria innocua* during its exponential and stationary growth phases. Our findings revealed the physiological changes associated with the application of this material, as observed using single-cell flow cytometry, photometric assays, as well as confocal and scanning electron microscopy. Both exponential- and stationary-phase *L. innocua* cells lost culturability after incubation with TF, but their cellular responses were different. After 30 min of contact with the TF, the nonculturable, stationary-phase cells maintained membrane integrity, membrane potential, stable respiratory electron-transport chains, and high levels of intracellular ATP. These phenotypic features, characteristic of the viable but nonculturable (VBNC) adaptation strategy, appear to be associated with upregulation of the stress-response factor *sigB*. In contrast, in TF-treated exponential-phase cells, membrane integrity was severely compromised and membrane potential showed hyperpolarization, suggesting sublethal injury accompanied by downregulation of *sigB*. Furthermore, the TF treatment enhanced the formation of extracellular vesicles in *L. innocua* and the deposition of extracellular polymeric substances in stationary-phase cells. The overall findings of this work demonstrate that sensitivity of *Listeria* cells to nature-based antimicrobials depends on their growth phase and, therefore, modulating their growth-phase-associated physiological status may counteract their VBNC defense strategy and improve biocidal efficacy.

**IMPORTANCE:**

In this work, we examined the efficacy of our novel, nature-based green sanitizer as a means to improve microbiological food safety without health and environmental risks, using gram-positive *Listeria innocua* as a model organism. *L. innocua* serves as a surrogate of the foodborne pathogen *L. monocytogenes* for evaluating sanitation efficacy and may also be involved in virulence and multidrug resistance transfer between the *Listeria* species. We demonstrate that the sanitizer effect depends on the physiological state of bacterial cells, which is affected by their growth phase. The treatment caused membrane damage to *L. innocua* in its exponential phase but induced the viable but nonculturable state during its stationary phase. Thus, this study demonstrates a way to overcome bacterial defense mechanisms and improve sanitizer efficacy by modulating the growth-phase-associated physiological status of targeted bacteria.

## INTRODUCTION

Ensuring the safety of foods, particularly raw and minimally processed fresh produce, is a critical challenge, as these foods may harbor different human pathogens (1). The genus *Listeria* is made up of gram-positive, facultative anaerobic, non-sporulating, rod-shaped bacteria commonly found in natural and industrial environments. *L. monocytogenes* is a hazardous pathogenic bacterium linked to an increasing number of outbreaks of foodborne disease (2, 3). *L. innocua* is closely related to *L. monocytogenes* and is often used as its surrogate for developing listericidal technologies and evaluating sanitation efficacy, as well as an indicator of potential contamination (4, 5). Furthermore, while *L. innocua* is usually considered a non-pathogenic species, it harbors a range of virulence genes and, under certain conditions, can cause human and animal diseases (6, 7). Even more importantly, *L. innocua* food isolates have been shown to contain numerous disinfectant- and antibiotic-resistance genes, including multidrug-resistance factors, and may serve as a reservoir for virulence and resistance genes that can be horizontally transferred between different *Listeria* species (8). Therefore, eradication of *L. innocua* should be an essential part of food-safety practices.

*Listeria* species are tolerant of adverse environmental factors and capable of forming biofilms. Despite routine sanitation, these microorganisms have a remarkable ability to survive in food-processing environments. While the industry commonly uses synthetic disinfectants, such as chlorine derivatives, to mitigate contamination, those materials often pose health and environmental concerns (9), and microorganisms may develop resistance or tolerance (10, 11). Therefore, there is an urgent need to develop novel, safe, and effective food sanitizers. Natural antimicrobials, such as phenolic compounds, are promising green alternatives to conventional chemicals for promoting microbiological food safety (12–14). However, the inferior antimicrobial potency of natural compounds in comparison with synthetic chemicals may limit their potential use (15). To enhance the microbiocidal efficacy of nature-based antimicrobials, researchers have explored their synergistic combinations (16–18). While bacteria can develop resistance to individual natural antimicrobials, similar to synthetic ones (19), the development of resistance to synergistic combinations of active principles with diverse modes of action and multiple cellular targets seems less likely or, at least, would take a longer time (20).

Our previous work demonstrated that the combination of the natural phenolic compound gallic acid (GA) with generally recognized as safe hydrogen peroxide (H_2_O_2_) possesses synergistic antimicrobial potency associated with the generation of reactive oxygen species (ROS), in particular, hydroxyl radicals (21), similar to the antimicrobial effect of photooxidized GA (22, 23). The GA + H_2_O_2_ combination has been found to be bactericidal toward various gram-negative species and gram-positive *Bacillus subtilis*, but has no significant effect on *L. innocua*, most probably due to *Listeria*’s high tolerance for different types of stress, including oxidative stress (24). Amending the GA + H_2_O_2_ combination with lactic acid (LA) yielded a triple formulation (TF) that allowed the synergistic inhibition of *L. innocua* proliferation without any additional increase in ROS generation (21). This synergy might be related to the enhancement of bacterial sensitivity to oxidative stress by acids, demonstrated in particular with *Listeria* (25, 26). While different organic acids have been found to potentiate the GA + H_2_O_2_ combination, lactic acid was associated with the highest level of activity, apparently beyond the pH effect (Fig. S1). The observed synergy could also be due to the permeabilizing effect of lactic acid (27). However, in spite of the growth inhibition, the TF-treated *L. innocua* cells were found to retain their viability, in agreement with the viable but nonculturable (VBNC) behavior pattern (21).

While the TF was developed and primarily tested with *L. innocua*, its efficacy against *L. monocytogenes* is of significant interest. In decontamination trials with pathogen-inoculated baby spinach, TF exhibited partial inhibition of *L. monocytogenes* that exceeded the control provided by commercial disinfectants such as peroxyacetic acid (28). It is plausible that VBNC induction was involved in this inhibition, as has been demonstrated for peroxyacetic acid (29). The effect of TF on *L. monocytogenes* deserves additional investigation and may demand further optimization of the formulation due to the enhanced resistance of this species to oxidative stress associated, in particular, with the pathogenicity factor PrfA (30).

The VBNC state is one of the adaptive survival mechanisms of non-spore-forming bacteria (31, 32). The VBNC bacteria can exhibit increased ability to withstand antimicrobials, including antibiotics (31, 33), which poses an obvious public health risk, especially considering the potential of VBNC pathogens to resuscitate and to regain virulence (34). The VBNC phenomenon highlights the limitations of standard, culture-based tests of disinfectant efficacy, which may lead to false-negative results. Modern, efficient molecular (35) and microscopic (36) protocols have been developed to detect the VBNC bacteria in decontamination systems.

Cellular mechanisms underlying the VBNC-induced TF effect on *Listeria* demand further investigation in order to improve the green sanitizer’s efficacy. A detailed definition of VBNC has been proposed by Liu et al. (37). Phenotypic features of VBNC include cell-division arrest combined with the maintenance of membrane integrity, intact membrane potential, and respiratory activity depending on a functioning electron-transport chain that enables ATP production (33). ATP generation is a reliable indicator of metabolic activity in microorganisms (38), including VBNC bacteria (39). In general, the VBNC state is characterized by the maintenance of a relatively high ATP level (40) that may be a survival mechanism to cope with an adverse environment (41). At the same time, some studies have associated the induction of a VBNC state with ATP depletion (42), while an increase in the ATP level has been shown to promote resuscitation (43). The morphological changes associated with the VBNC state include cell-size reduction and shift to a coccoidal shape (44), probably as a strategy to minimize energy requirements (34).

The cellular and molecular mechanisms of VBNC induction are not completely clear. ROS and antioxidant factors are presumably involved in VBNC induction (40, 45). In gram-positive bacteria, such as *Listeria*, the transition to a VBNC state is modulated by the stress-response regulator SigB, although VBNC can be induced in *sigB*-deficient *Listeria* mutants (46, 47). Various stress-response factors, such as the stringent response (46, 48), SOS response (49, 50), the toxin-antitoxin system (45, 48), or their combinations (51, 52) have been suggested to regulate VBNC induction. In particular, bacterial cell division is suppressed by the SOS response system, which contributes to DNA repair in stressed cells and involves the cell-division inhibitor YneA (53).

The propensity of bacteria to enter a VBNC state depends on their physiological state, particularly their growth phase. In the stationary phase, bacteria often show greater stress resistance (54, 55) and viability under adverse conditions than they do during the exponential phase (45, 46). *Listeria* cells entering the stationary phase undergo adaptation that ensures their survival (56) as it provides them with improved tolerance of heat (57), acidity (58), and natural antimicrobials (59). The improved fitness of stationary-phase *Listeria* has been associated with enhanced expression of the stress-response regulator SigB and the antioxidant enzyme catalase (59, 60). At the same time, Bortolussi et al. ((61)) reported that stationary-phase *L. monocytogenes* was apparently less resistant to ROS than exponential-phase *L. monocytogenes*.

In this work, we aimed to characterize the cellular response of *L. innocua* to a green antimicrobial formulation, as affected by the growth phase. Major cellular responses were found to be maintained differently among the stationary-phase cells than among the exponential-phase cells, and the VBNC adaptation strategy of the stationary-phase cells was shown to involve upregulation of the stress-response factor *sigB*.

## MATERIAL AND METHODS

### Culture preparation

Briefly, a typical colony of *Listeria innocua* ATCC 33090 from an overnight-grown plate was inoculated into 20 mL of Brain Heart Infusion (BHI) broth (HiMedia Laboratories Pvt Ltd., Thane, India) and incubated at 37 ± 1°C with continuous shaking at 180 rpm. The bacterial growth was monitored by hourly measurements of the optical density at 600 nm (OD_600_) of 200 µL of culture, using a spectrophotometer, GENESYS 10S UV-Vis (Thermo Fisher Scientific, Waltham, MA, USA), for 24 h. The data were logarithmically transformed to log_10_ OD_600_ and plotted against time to construct growth curves. The exponential growth phase was identified as a linear increase portion of the transformed optical density curve, while the stationary phase was identified as a horizontal portion of the curve (62, 63). For the experiments, cultures were collected after 8 h (exponential) or 16 h (stationary) of growth (Fig. S2). The cells were harvested by centrifugation, and the pellet was resuspended in 0.85% (wt/vol) saline solution. The suspensions were then adjusted to reach approximately 10^9^ CFU mL^−1^ with reference to OD_600_ and used as inocula for testing the effects of the TF.

### TF treatment and evaluation of culturability

The inocula were brought to a cell density of 10^8^ CFU mL^−1^ with the saline-based TF mix comprising 8 mM GA, 1 mM H_2_O_2_, and 20 mM LA, for a final volume of 75 mL in 250 mL Erlenmeyer flasks. The TF ingredients were purchased from Sigma-Aldrich. Saline solution without the TF ingredients was used as a control. The cell suspensions were incubated at 22 ± 1°C on an orbital shaker (Lab-Line Instruments, Inc., Melrose Park, IL, USA) at 200 rpm. The duration of incubation varied from 7 to 30 min in initial trials and was subsequently standardized as 30 min. After the incubation, the cells were collected under vacuum on 0.2 μm filters (Tamar Laboratory Supplies Ltd., Mevaseret Zion, Israel), thoroughly washed, and resuspended in saline solution, followed by the culturability and viability assessments described below. Aliquots (100 µL) of the samples were serially diluted, plated on BHI agar, and cultured at 37°C ± 1°C for 24 h. The culturability of the TF-treated and control samples was expressed as the number of culturable cells (log CFU mL^−1^) recovered from the initial 10^8^ CFU mL^−1^ inoculum.

### Membrane-integrity assay

TF-treated and untreated samples were concentrated and examined using the LIVE/DEAD BacLight Bacterial Viability Kit (Molecular Probes, Invitrogen, Eugene, OR, USA) according to the manufacturer’s instructions. This assay utilizes SYTO 9 and propidium iodide (PI) fluorescent dyes with varying spectral characteristics. SYTO 9 (green fluorescence) labels the DNA of both live and dead bacteria, while PI (red fluorescence) only penetrates membrane-compromised cells (64). As a result, cells with intact membranes emit green fluorescence, and membrane-compromised cells emit orange to red fluorescence. The samples (100 µL) were mixed with equal volumes of the dual stain and incubated for 15 min in the dark, after which fluorescence was measured at 530 nm (green, G) and 630 nm (red, R). Relative proportions of viable cells after TF treatment were calculated from G/R ratios, and data were expressed as percentages of untreated cells (considered as 100%) from the same growth phase.

### Flow cytometry (FCM) analysis

Filtered samples (40 μm) were incubated with different dyes in black, 96-well plates. Fluorescence was excited by a 488 nm laser and measured using default filters of the FL1 (FITC) and FL3 (PERCP-A) channel for green (530/20 nm) and red fluorescence (630/20 nm) optical bandpasses, respectively, depending on the dye, using a flow cytometer, BD Accuri C6 Plus (BD Life Sciences, San Jose, CA, 95131, USA). The analysis was conducted at a medium flow rate of 35 μL min^−1^, 16 μm core for approximately 150 μL of sample and a threshold of 10,000 events. The data were analyzed using FlowJo v.10 software (BD Life Sciences). Untreated samples and heat-inactivated (85 ± 1°C, 10 min) samples in saline solution defined the gating strategy, while unstained samples facilitated the correction of background noise and excluded debris. Relative proportions of viable cells after TF treatment were calculated from R/G ratios (membrane potential) or absolute values (membrane integrity and electron transport), and data were expressed as percentages of untreated cells (considered as 100%) from the same growth phase.

#### FCM membrane-integrity assay

Bacterial staining with PI to detect membrane-compromised cells was carried out according to the manufacturer’s bacteria-viability manual (Molecular Probes, Invitrogen). Cells with compromised membranes were stained red with PI (64–66). Aliquots of the samples (200 μL) were incubated with 4 μL PI (final concentration, 30 µM), in a black, 96-well plate for 15 min in the dark.

#### FCM membrane-potential assay

Membrane potential was examined using a BacLight bacterial membrane potential kit (Molecular Probes, Invitrogen) based on the manufacturer’s instructions. In black, 96-well plates, treated and untreated cell suspensions (100 μL) were mixed with the DiOC_2_(3) dye (3,3′-diethyloxacarbocyanine iodide) in phosphate-buffered saline (PBS) solution with a final concentration of 30 μM and then incubated for 5 min in the dark. Ratios of green to red fluorescence were reported and compared with those observed for the untreated control or carbonyl cyanide m-chlorophenylhydrazone (CCCP) at a working concentration of 5 μM, which was used to prepare reference cells with depolarized membranes. Incubation with 1% D-glucose (Sigma-Aldrich) for 30 min at room temperature was used as a reference hyperpolarization treatment (67, 68).

#### FCM electron-transport assay

To measure electron-transport activity, samples were analyzed using 5-cyano-2,3-ditolyl tetrazolium salt (CTC) reagent. That procedure was performed as described by Silva et al.(69) (), with minor adjustments. Cell suspensions (200 µL) were incubated in PBS with 2.5 mM CTC (Biotium, Inc., Fremont, CA, USA) at 37°C for 1 h. In actively respiring, viable cells, the dye was rapidly reduced by the electron-transport chain to an insoluble formazan product, rendering the respiring (CTC-positive) cells fluorescent red (70, 71). Cells treated with the electron-transport disruptor sodium azide (Sigma-Aldrich Israel Ltd., Rehovot, Israel) were used as a negative control. These cells were incubated with 50 mM sodium azide for 30 min at 22°C.

### Measurement of ATP levels

The ATP levels produced by the cells were determined using the BacTiter-Glo^T^ (Promega Corp., Madison, WI, USA) kit according to the manufacturer’s protocol. This method is based on the reaction of luciferin and cellular ATP in the presence of Mg^2+^, O_2_, and luciferase enzyme to produce a glow-type signal registered as luminescent light. The luminescent signal correlates with the amount of ATP, which, in turn, reflects the number of viable cells in the sample. The reagent was reconstituted by transferring 10 mL of thawed buffer into a vial of the substrate and mixing to get a homogeneous solution equilibrated at room temperature before use. To get rid of extracellular ATP, samples were washed three times with saline solution. A volume of 100 μL of the bacterial suspension (10^8^ CFU mL^−1^) from each dilution was mixed and incubated with 100 μL of BacTiter-Glo reagent (1:1) in white, polystyrene, 96-well plates for 5 min. The luminescence values were observed using a BioTek Synergy 2 Microplate Reader (BioTek Instruments, Winooski, VT, USA). The relative number of viable cells after TF treatment was calculated from absolute ATP values, and data were expressed as percentages of untreated cells (considered as 100%) from the same growth phase.

### Confocal laser-scanning microscopy

As described previously, samples were stained with LIVE/DEAD BacLight stain. After incubation, 5 µL of cell suspension was transferred onto a glass slide and visualized under a Leica SP8 laser-scanning confocal microscope (Leica, Wetzlar, Germany) equipped with an HC PL APO 63×/1.20 water-immersion objective.

### Scanning electron microscopy (SEM)

Briefly, TF-treated and untreated cell pellets, in duplicate, were washed twice with PBS (pH 7.4) and then centrifuged at 500 × *g* for 5 min. For fixation, samples were incubated overnight with 1 mL of 2.5% glutaraldehyde (50% in water, Sigma-Aldrich) at 4°C, followed by three PBS wash/centrifugation cycles at 2,700 × *g* (7,000 rpm, 10 min), and then resuspended in 1 mL PBS. Samples (500 µL) were transferred onto 12 mm circular glass coverslips coated with poly-L-lysine solution (Sigma), placed in a 24-well plate for 1 h for attachment, and then dehydrated in an increasing gradient series of ethanol (10, 20, 30, 50, 70, 90, and 3 times 100%) with 5 min treatments and 5 min intervals between treatments, and finally dried in a critical point dryer (K850; Quorum Technologies Ltd., Loughton, UK). Samples were attached to a SEM stub using carbon tape and sputter-coated with gold-palladium using a SC7620 mini sputter coater (Quorum Technologies). The samples were examined with a TESCAN MIRA SEM with a field emission gun Schottky electron source operating at 5 kV accelerating voltage and using a TESCAN’s Essence software (TESCAN, Brno, Czech Republic). Fiji open-source image processing software (72) was used to capture cellular dimensions, length, and width (µm), across the central region of 30 random individual bacteria for each sample.

### Quantitative analysis of Sigma B encoding (*sigB*) gene expression by qPCR

Following TF treatment, total RNA was extracted using the Qiagen RNA isolation kit (QIAGEN N.V., Hilden, Germany) according to the manufacturer’s instructions. Briefly, freeze-dried pellets were enzymatically and mechanically lysed. RNA was purified using silica column-based extraction and treated with TURBO DNase I (Thermo Fisher Scientific Inc., Waltham, MA, USA) to remove genomic DNA. RNA concentration and purity were assessed using a NanoDrop spectrophotometer (Thermo Fisher Scientific, Inc.), and integrity was confirmed by agarose gel electrophoresis. Reverse transcription to synthesize 20 μL of cDNA was carried out using a high-capacity cDNA reverse-transcription kit (Thermo Fisher Scientific, Inc.). That procedure was performed at 37°C for 120 min and followed by enzyme inactivation at 85°C for 5 min. Using an Applied Biosystems 7500 thermal cycler (Thermo Fisher Scientific, Inc.), 10 μL qPCR reactions containing 5 μL 2× Fast SYBR Green Master Mix (Thermo Fisher Scientific, Inc.), 0.5 μL of each primer (0.5 μM), 2 μL template DNA, and 2 μL DNase-free water were performed under the following thermal cycling conditions: 95°C, 20 s for activation 40 cycles of 95°C, 3 s for denaturing, 60°C, and 30 s for annealing/extension. s*igB* expression was quantified using (F-GATGATGGATTTGAACGTGTGAA, R-CGCTCATCTAAAACAGGGAGAAC) primers designed in this study. The cycle thresholds were normalized with the expression ratio of 16S rRNA (F-GCTAACTACGTGCCAGCAGC, R-GCACTCCAGTCTTCCAGTTTCCA) used as a reference gene. The changes were calculated as relative expression by the critical threshold method described by reference 73 according to the equation:


$$\begin{array}{c} \Delta \Delta C_{T}={\left(C_{T,\text{ Target }}-C_{T,\text{ Reference }}\right)}_{\text{Experimental sample }}-{\left(C_{T,\text{ Target }}-C_{T,\text{ Reference }}\right)}_{\text{ Untreated control,}}\text{and}\\ \text{ Fold Change }=2^{-\Delta \Delta C\;T}. \end{array}$$

### Statistical analysis

All of the trials were performed in triplicate, unless specified otherwise. For statistical analysis, the microbiological data were transformed into logarithmic form as decimal logarithms of the CFU and expressed as means within a *t*-based confidence interval at a 95% confidence level. Statistical significance of the differences between the means was determined by one-way ANOVA and compared using pairwise Tukey’s HSD tests (*P* < 0.05) performed using JMP 15 statistical software (SAS Institute, Inc., Cary, NC, USA).

## RESULTS

### Characterizing effects on culturability

**Fig 1:**
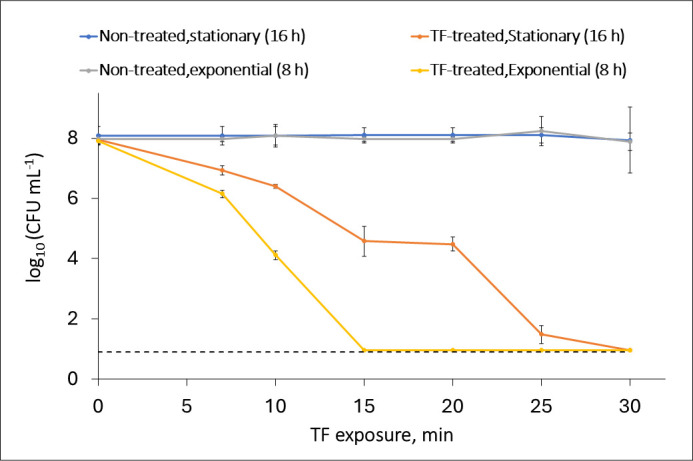
Effects of the duration of TF exposure and inoculum growth phase on the culturability of *L. innocua* on BHI at 22 ± 1°C. Untreated control: incubation in saline solution without TF. Each value indicates the mean result of tests performed in duplicate. A dotted black line indicates the limit of detection. Error bars represent 95% confidence intervals (*P* < 0.05).

We found that the inoculum growth phase significantly influenced the response of *L. innocua* to TF. The high-density (8 log CFU mL^−1^) suspension of exponential-phase cells lost culturability to undetectable levels after 15 min of contact with TF. In comparison, among the stationary-phase cells, culturability was lost after 30 min of incubation (Fig. 1). In previous research, we found that lower-density (5 log CFU mL^−1^) stationary-phase *L. innocua* cell suspensions lost culturability after just 5 min of contact with TF (Fig. S3) (74).

### Membrane integrity

**Fig 2:**
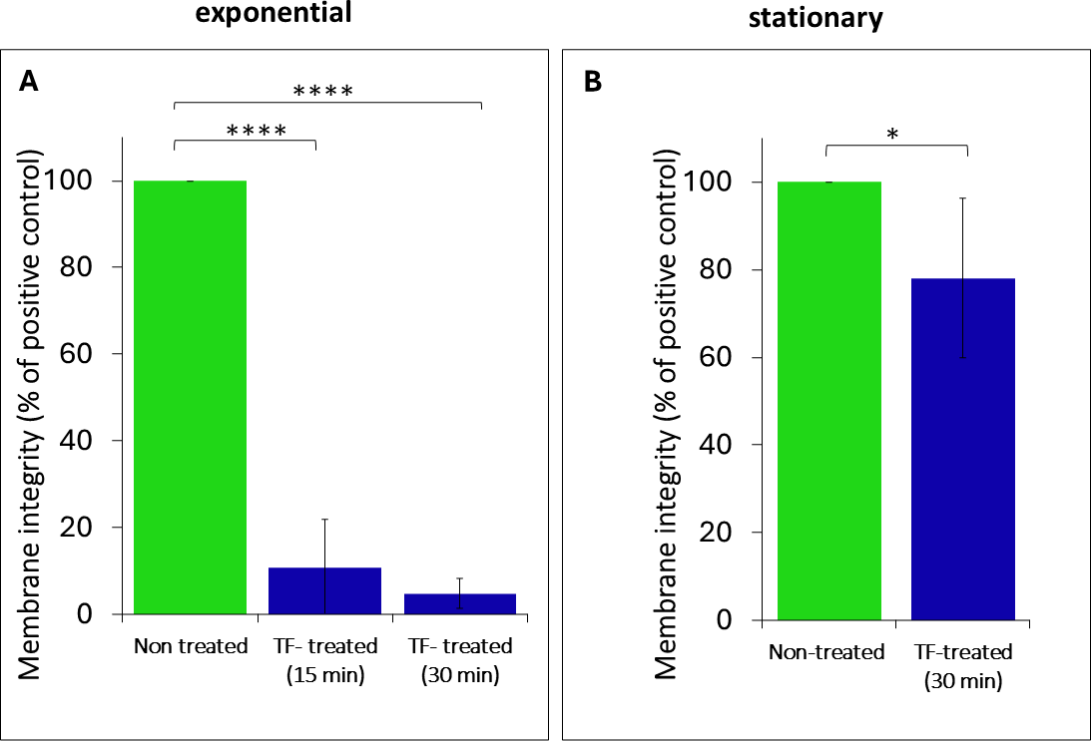
Effects of TF treatment on the viability of nonculturable (**A**) exponential-phase and (**B**) stationary-phase cells, as determined using a LIVE/DEAD assay, which measured the retention of membrane integrity relative to the untreated control. The duration of TF exposure was 15 or 30 min for exponential-phase cells and 30 min for stationary-phase cells. Data represent mean results of duplicate tests. Error bars represent 95% confidence intervals (*P* < 0.05). Asterisks indicate significant differences relative to the untreated control (****P < 0.0001; *P < 0.05).

While cells from both the exponential and stationary phases lost culturability following TF treatment, the LIVE/DEAD BacLight assay revealed a marked difference in their membrane integrity. Despite the loss of culturability, the stationary-phase cells maintained their membrane integrity after 30 min of exposure to TF, as shown in Fig. 2B. In contrast, the exponential-phase cells showed significant membrane damage following both 15 and 30 min of the TF exposure (Fig. 2A). In further experiments, we applied a uniform 30 min TF exposure for both exponential- and stationary-phase cells.

**Fig 3:**
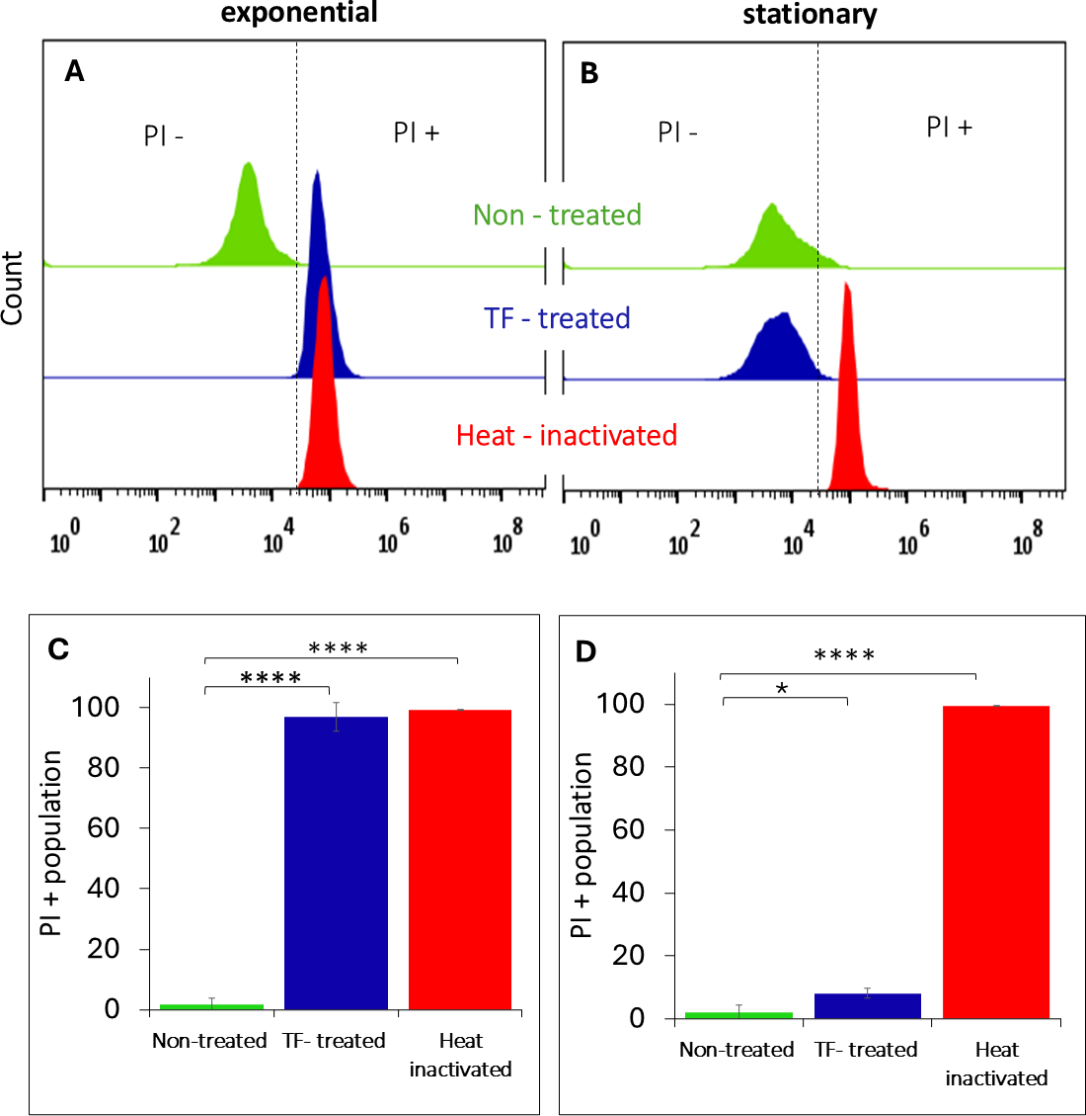
Flow cytometry half-offset histograms of PI-stained (**A**) exponential-phase and (**B**) stationary-phase cells treated with TF, in comparison with untreated and heat-inactivated controls. Effects of TF treatment on the percentage of PI-positive (PI+) cells among (**C**) exponential- and (**D**) stationary-phase cell populations, in comparison with untreated and heat-inactivated controls. Data represent mean results of tests performed in triplicate. Error bars represent 95% confidence intervals (*P* < 0.05). Asterisks indicate significant differences relative to the untreated control (****P < 0.0001; *P < 0.05).

Flow cytometry analysis revealed that PI stain profoundly penetrated >99% of TF-treated exponential-phase cells, indicative of compromised membrane integrity (Fig. 3A and C), similar to the bacteria subjected to heat inactivation at 85°C. Conversely, more than 90% (Fig. 3D) of the TF-treated stationary-phase cells remained PI-negative, similar to the distribution observed for the untreated control and indicating the maintenance of membrane integrity (Fig. 3B and D). Interestingly, membrane integrity was also maintained among the stationary-phase *L. innocua* cells after 10 min of exposure to 70°C heat (Fig. S4).

**Fig 4:**
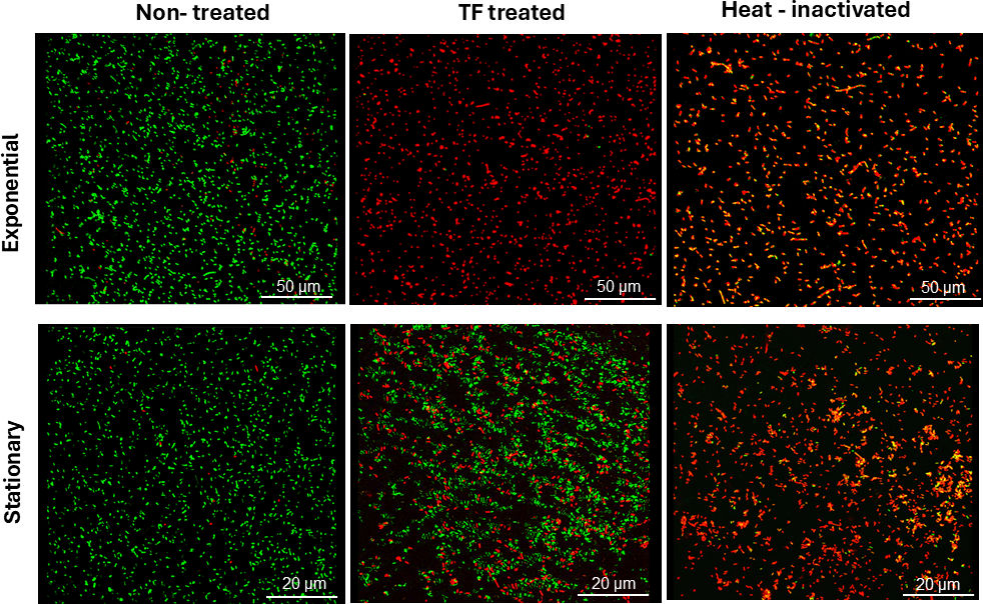
Microscopic images of LIVE/DEAD BacLight-stained exponential-phase and stationary-phase *L. innocua* cells: untreated control, TF-treated, and heat-inactivated.

These findings were corroborated by a molecular PMAxx assay (Fig. S5) and a microscopic study using dual staining together with the LIVE/DEAD BacLight assay (Fig. 4). Red fluorescence indicated the penetration of PI through the damaged membranes of TF-treated exponential-phase cells, similar to heat-inactivated (85°C) ones. The intactness of membranes in untreated and TF-treated stationary-phase cells was confirmed by the absence of PI and green fluorescence associated with the SYTO 9 dye.

### Membrane potential

**Fig 5:**
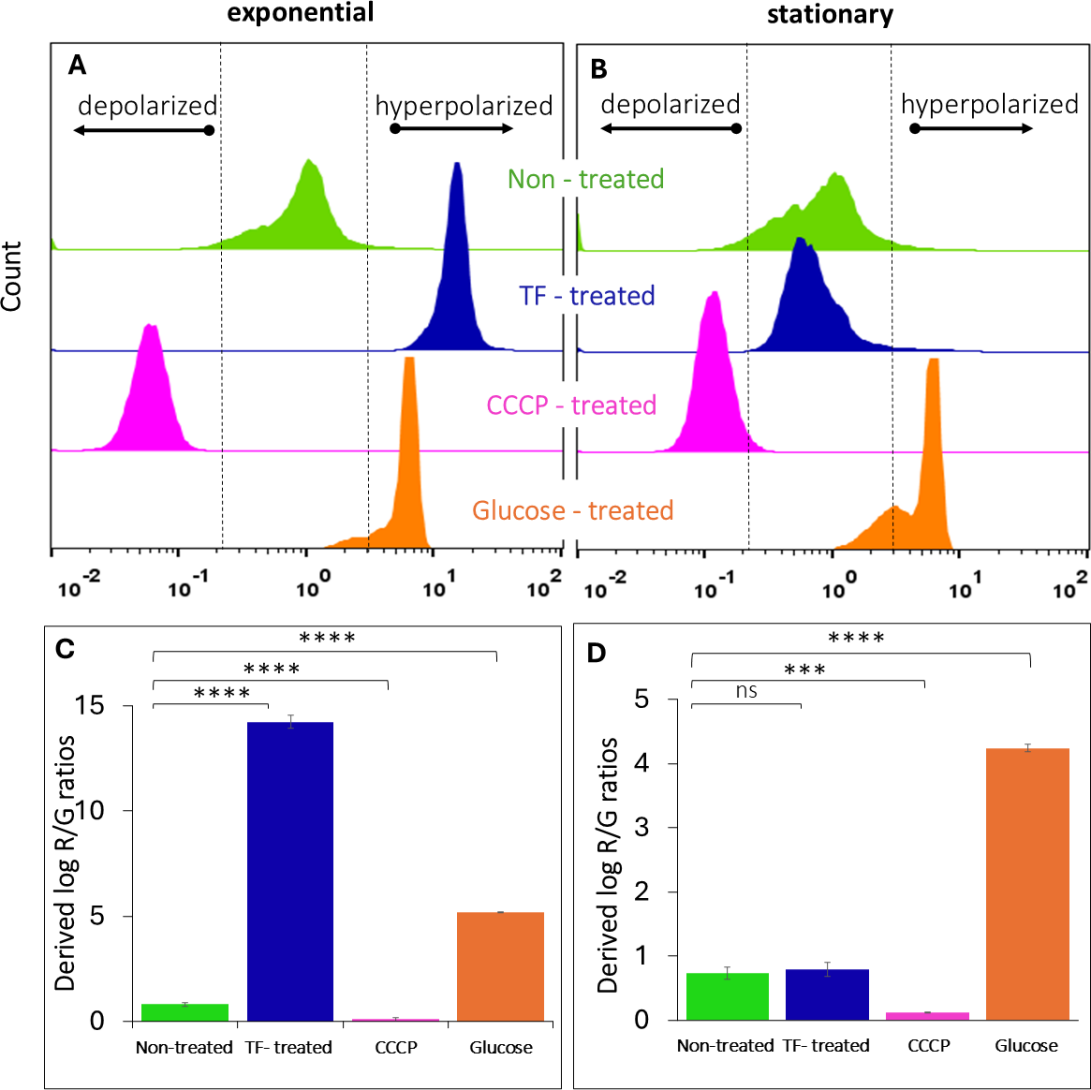
Flow cytometry half-offset histograms of DiOC_2_(3)-stained (**A**) exponential-phase and (**B**) stationary-phase cells treated with TF, in comparison with untreated, CCCP-treated, and glucose-treated controls. Effects of TF treatment on the membrane potential shifts presented as log-transformed red/green fluorescence ratios of (**C**) exponential-phase and (**D**) stationary-phase cells, in comparison with untreated, CCCP-treated, and glucose-treated controls. Data represent mean results of tests performed in duplicate. Error bars represent 95% confidence intervals (*P* < 0.05). Asterisks indicate significant differences relative to the control (****P < 0.0001; ***P < 0.001; ns, non-significant).

In Fig. 5, we present the effect of TF on the membrane potential of exponential- and stationary-phase cells, in comparison with untreated cells and cells treated with depolarization (CCCP) and hyperpolarization (glucose) agents, as investigated using the DiOC_2_(3) probe. Among exponential-phase cells, the TF treatment resulted in a notable upshift in red fluorescence, indicating membrane hyperpolarization (Fig. 5A and C). However, TF did not affect the membrane potential of the stationary-phase cells, which showed a membrane potential distribution similar to that of the untreated cells (Fig. 5B and D).

### Membrane electron transport

**Fig 6:**
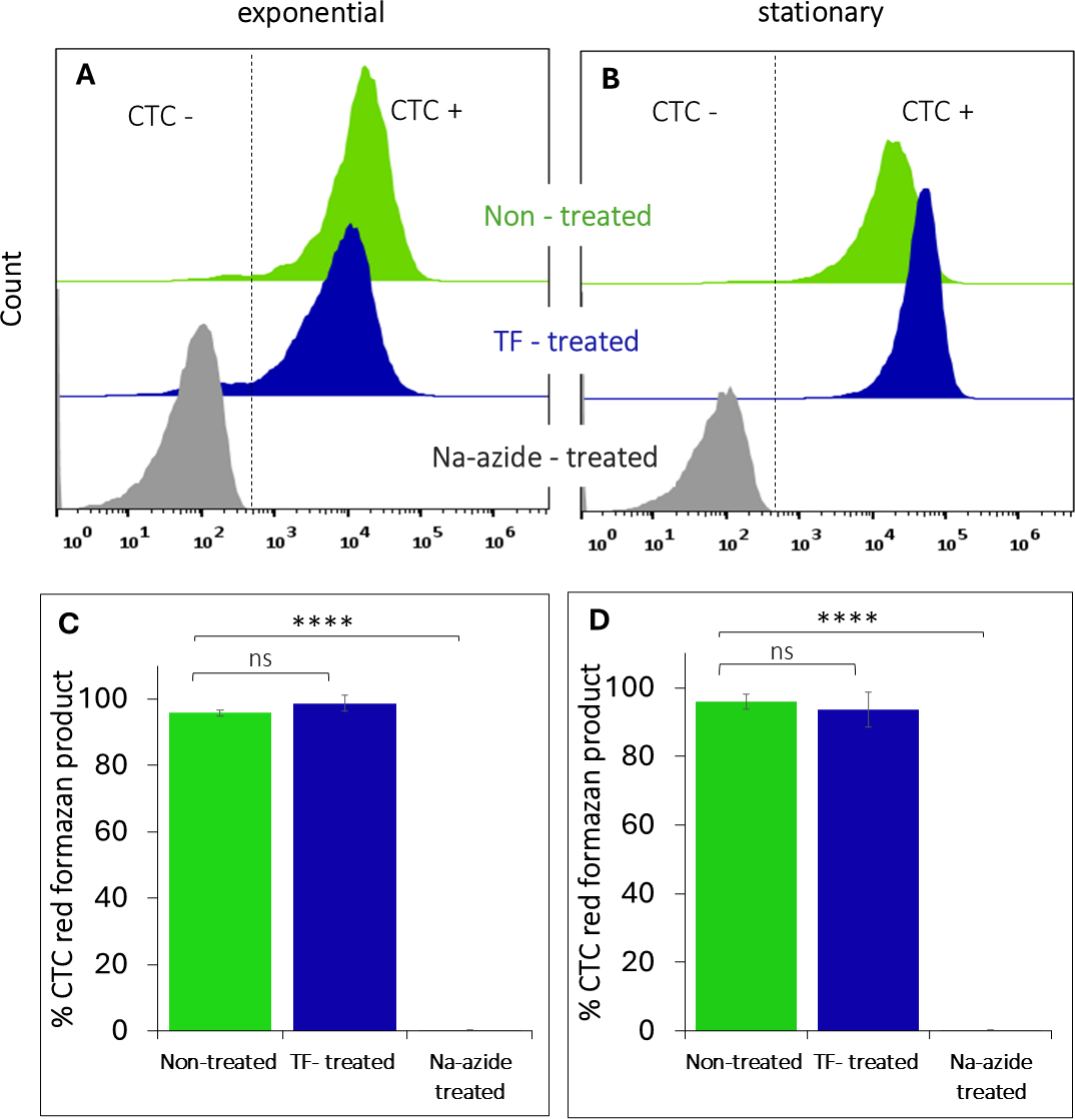
Flow cytometry half-offset histograms of CTC-stained (**A**) exponential-phase and (**B**) stationary-phase cells in comparison with untreated and sodium azide-treated controls. Effects of TF treatment on the percentage of CTC-positive (CTC+) cells in (**C**) exponential- and (**D**) stationary-phase cell populations, in comparison with untreated and sodium azide-treated controls. Data represent mean results of tests performed in triplicate. Error bars represent 95% confidence intervals (*P* < 0.05). Asterisks indicate significant differences relative to the control (****P < 0.0001, ns, non-significant).

The results of the CTC assay are presented in Fig. 6. According to the assay results, the TF treatment had a minor effect on the respiratory electron-transport system in both exponential- and stationary-phase bacteria, in contrast to cells treated with sodium azide, a known electron-transport chain disruptor.

### Intracellular ATP levels

**Fig 7:**
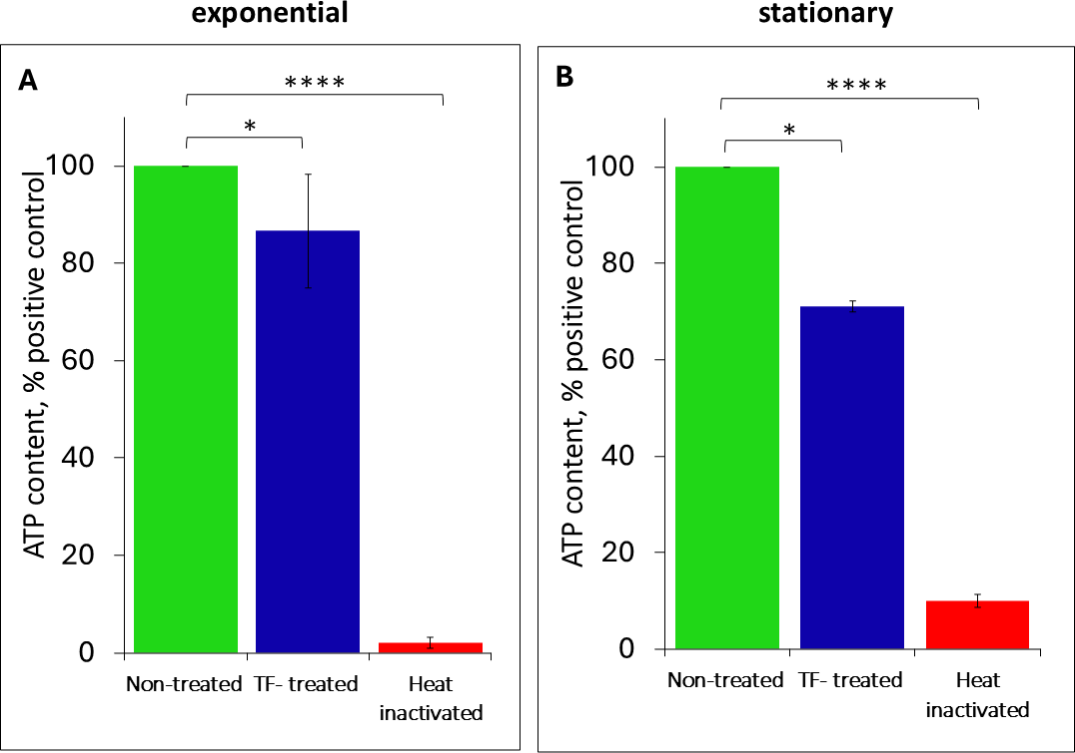
Effects of TF treatment and heat inactivation on intracellular ATP levels among (**A**) exponential-phase and (**B**) stationary-phase cells relative (%) to the untreated control, as determined using a BacTiter-Glo assay. Error bars represent 95% confidence intervals (*P* < 0.05). Asterisks indicate significant differences relative to the control (****P < 0.0001; *P < 0.05).

We next measured relative intracellular ATP levels as a reflection of cellular energy metabolism. In agreement with the maintenance of respiratory metabolic activity, following TF exposure, both exponential- and stationary-phase bacteria demonstrated rather high intracellular ATP levels, close to those observed among the untreated cells (Fig. 7A and B). In contrast, very little ATP was detected in the heat-inactivated bacteria.

### Cellular morphology and gene expression

**Fig 8:**
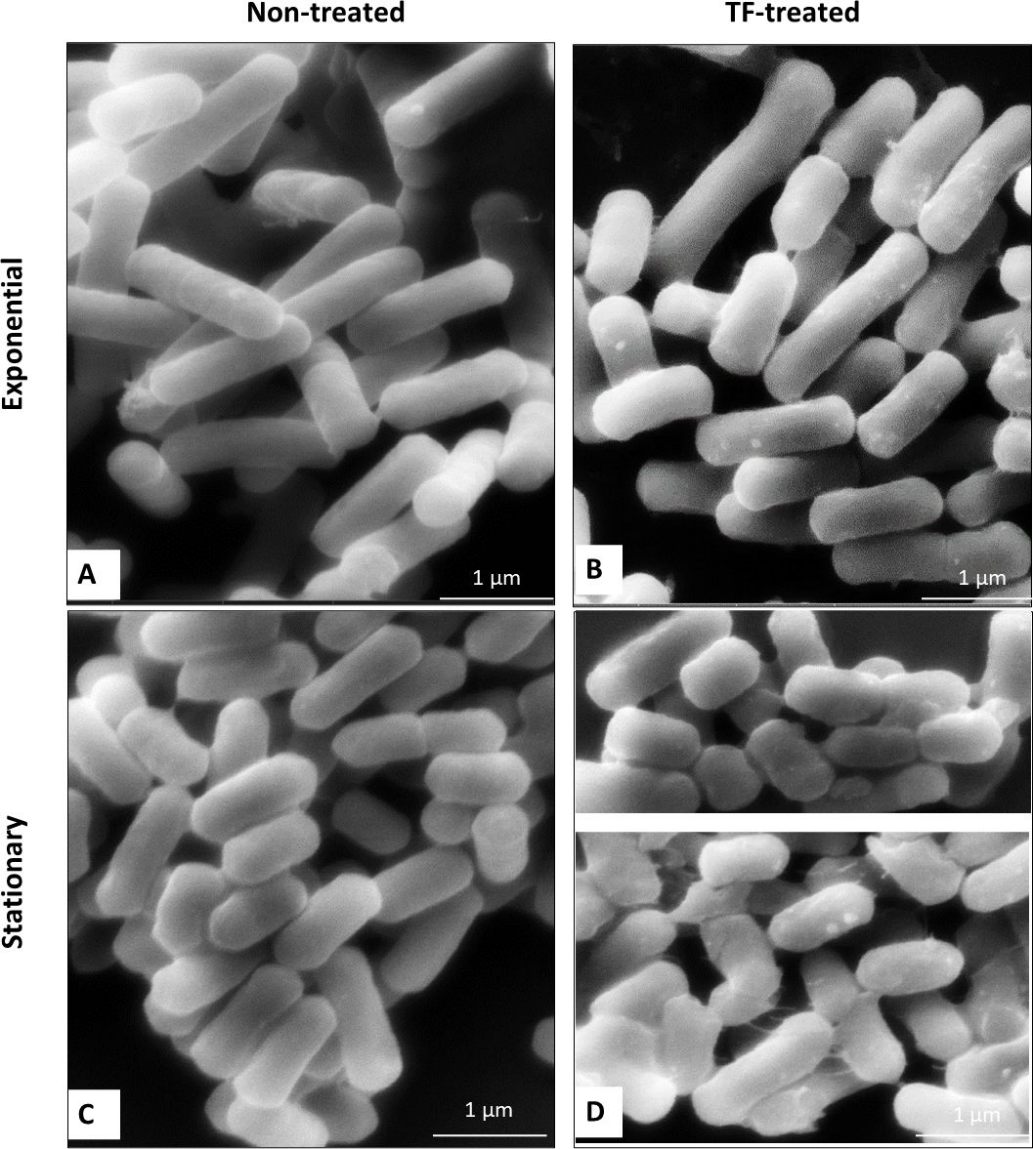
Scanning electron micrographs of *L. innocua* cells in the exponential phase (**A**, untreated;** B**, TF-treated) and stationary phase (**C**, untreated; **D**, TF-treated).

We further analyzed morphological changes associated with TF treatment through SEM visualization. SEM micrographs of the *L. innocua* cells in two growth phases, treated with TF or untreated, are presented in Fig. 8.

The shift from the exponential to the stationary phase resulted in significant cell shortening and a certain increase in cell width (Table 1). The SEM examination did not detect any significant dimension changes or visible cellular damage caused by the TF treatment, irrespective of the growth phase. At the same time, the TF-treated cells displayed frequent protruding structures of approximately 70 nm–100 nm in diameter, presumably extracellular vesicles. In addition, the TF-treated stationary-phase cells were often interconnected by deposits or, presumably, filaments of extracellular polymeric substances (EPS).

**TABLE 1:** Effects of growth phase and TF treatment on the dimensions of *L. innocua* cells^*a*^

Growth phase	Treatment	Length (µm)	Width (µm)
Exponential	Untreated	1.155 ± 0.135^a^	0.347 ± 0.007^b^
	TF-treated	1.067 ± 0.102^a^	0.353 ± 0.006^b^
Stationary	Untreated	0.782 ± 0.069^b^	0.377 ± 0.011^a^
	TF-treated	0.816 ± 0.076^b^	0.379 ± 0.013^a^

^*a*^ Values represent means of 30 random individual cells ± 95% confidence intervals (*P* < 0.05). Different letters indicate significant differences between values in columns, according to Tukey’s HSD test (*P* < 0.05).

**Fig 9:**
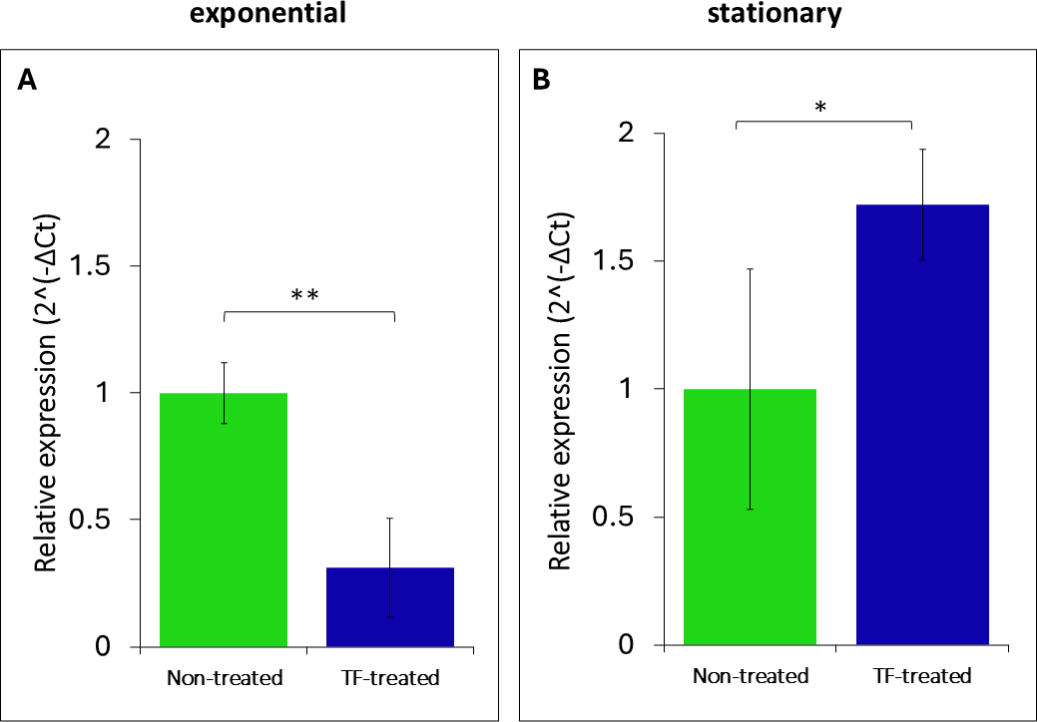
Normalized relative expression of the Sigma B (SigB)-encoding gene after TF treatment of (**A**) exponential-phase and (**B**) stationary-phase *L. innocua* cells. Data represent mean results of tests performed in triplicate. Error bars represent 95% confidence intervals (*P* < 0.05). Asterisks indicate significant differences relative to the control (**P < 0.01; *P < 0.05).

The TF treatment tended to upregulate the expression of the *sigB* gene, indicating an inducible stress response (Fig. 9). At the same time, *sigB* expression in the TF-treated exponential-phase cells was significantly downregulated compared to the untreated control.

## DISCUSSION

The findings of this study suggest that the response of stationary-phase *L. innocua* cells to TF follows the VBNC pattern, in agreement with our previous work (21). After 30 min of contact with the TF, the stationary-phase cells lost their culturability but maintained their membrane integrity, membrane potential, and metabolic activity (manifested as stable electron-transport-chain activity and a high level of intracellular ATP), all phenotypic features characteristic of the stress-induced, adaptive VBNC mechanism (36). In agreement with this interpretation, the TF treatment was found to upregulate the stress-response *sigB* factor in the stationary-phase cells. These findings align with the literature showing VBNC induction by various sanitizers (28, 34). They demonstrate that purely growth-based methods are insufficient for testing antimicrobial agents and should be reevaluated and amended with sensitive VBNC-detecting techniques.

In contrast to previous reports (44, 75), there was no significant difference between the shape of the TF-induced VBNC cells and that of the untreated cells of the same growth phase. This similarity is not surprising, considering the brief TF exposure and the short time elapsed between the treatment and the cell sampling. Enhanced extracellular polymer (EPS) production, resulting in cell clumping (76), as well as the formation of extracellular vesicles (77), was observed in VBNC *S. aureus* cells after nonthermal plasma treatment. Interestingly, in *Listeria*, the formation of extracellular vesicles is controlled by the *sigB* factor and associated with bacterial survival under stressful conditions (78). The enhanced EPS production observed among the TF-treated *L. innocua* cells might foster their aggregation and surface attachment. However, it is less probable that the VBNC-inducing TF treatment could stimulate mature biofilm formation because further stages of biofilm growth are typically associated with cell division (79).

In contrast, among the exponential-phase cells, the TF treatment resulted in compromised membrane integrity and membrane hyperpolarization, accompanied by *sigB* downregulation. Similar phenomena were observed in *L. monocytogenes* treated with another phenolic compound, quercetin, that caused membrane damage, repressed *sigB* and virulence genes, and inhibited biofilm development (80). Maintenance of membrane integrity is one of the key criteria of cell viability (35, 81), so these cells could not be defined as truly viable. In many studies, membrane hyperpolarization caused by various antimicrobial agents has been associated with subsequent cell death among both gram-negative and gram-positive bacteria (82–84). For example, the oxidative stress caused to *Pseudomonas aeruginosa* and *Bacillus subtilis* by blue light has been shown to result in hyperpolarization followed by depolarization to a level below the initial resting potential and cell death (84). Similar ROS generation, oxidative stress, membrane damage, and hyperpolarization were caused by CO_2_ in a *Shewanella putrifaciens* culture (85).

Despite the apparent membrane damage and hyperpolarization, the TF-treated exponential-phase cells demonstrated cellular metabolic activity, manifested in the CTC assay as functioning respiratory electron-transport chains. Although the CTC reduction might be caused by superoxide radicals as opposed to electron-transfer enzymes (86), the concomitant ATP accumulation was evidence of the true respiratory activity of the TF-treated exponential cells (87). Similar phenomena have been observed in electromagnetic field-treated *E. coli*, *S. aureus*, and *Pseudomonas putida* (88, 89). Such a state suits the definition of sublethal injury (i.e., the situation in which the bacterial membrane is damaged, but the metabolic activity is still present). In *L. monocytogenes*, sublethal injury was induced by grape seed extract rich in phenolic compounds, which caused membrane damage and metabolic disorders in bacterial cells (90).

Sublethally injured cells can subsequently move into a dead or alive state (91). We assume that the former direction might prevail in the TF-treated exponential-phase *L. innocua*. Maintenance of electron transport and ATP accumulation in these cells, in combination with membrane damage and hyperpolarization, could be a part of cellular degradation processes. For example, altered ATP flux and ATPase activity (88, 92), as well as accelerated electron-transport-chain activity (93), were reported to result in oxidative stress, membrane damage, hyperpolarization, and, eventually, voltage-induced death. Notably, in this situation, applying the depolarization agent CCCP and/or ATPase inhibitor improved cell survival (94).

The findings of this study demonstrate the higher propensity of stress-challenged stationary-phase cells to enter the adaptive VBNC state, as compared to exponential-phase cells. The shift from the exponential phase to the stationary phase is characterized by drastic changes in cell structure and function, which are driven by upregulation of global regulatory responses (95). Yet in the 1990s, it was established that stationary-phase-regulating sigma transcription factors, such as RpoS (sigmaS) for gram-negative bacteria, are also responsible for bacterial stress responses (96). In gram-positive bacteria, such as *Listeria*, a range of sigma factors, in particular *sigB*, are upregulated during the stationary phase (55) and determine the general stress response controlling defense against various adverse factors (95). In *Listeria*, the *sigB* factor contributes to long-term stationary-phase survival and influences the development of the growth advantage in stationary phase phenotype (97). We observed upregulation of the *sigB* gene expression in stationary-phase cells after TF treatment that was associated with its inducible stress response.

There is a certain similarity between the cellular processes bringing the bacteria to the stationary growth phase and those involved in the development of the VBNC state. The adaptations that take place in bacterial cells during the late stationary phase predispose them to enter the VBNC state (55). Furthermore, Ughy et al. (98) suggested that the late stationary phase itself can be considered a VBNC state, being an inevitable genetically programmed stage of bacterial population evolution. However, the difference between the late stationary phase and the true VBNC cells, as defined by Liu et al. (37), lies in the ease of resuscitation. Stationary-phase cells normally regain culturability and undergo outgrowth just after transfer to a fresh nutrient medium (99), while true VBNC bacteria need special conditions for resuscitation (43). Sodium pyruvate has been proposed as a potential resuscitation factor for *Listeria* (46), with encouraging preliminary results in our trial (Fig. S6).

**Fig 10:**
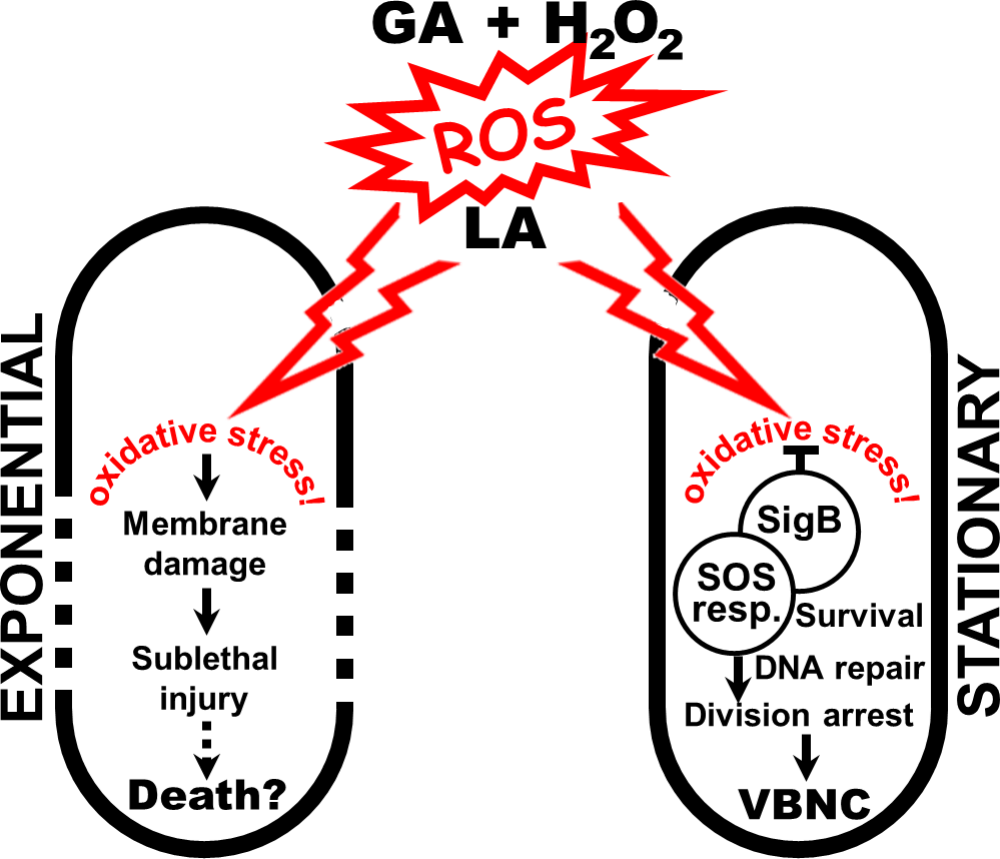
Schematic representation of proposed mode of action of the TF. Interaction of GA with H_2_O_2_ generates ROS, causing oxidative stress in *L. innocua* cells in a manner mediated by LA. Under this stress, vulnerable exponential-phase cells suffer membrane damage and sublethal injury that may progress to cell death. In contrast, stationary-phase cells survive the stress through *sigB*-mediated defense, presumably via a DNA-repairing SOS response that leads to arrested cell division and subsequent VBNC. Dotted and solid cell outlines represent compromised and intact membranes, respectively.

A hypothetical scheme summarizing our interpretation of the TF effects on exponential- and stationary-phase cells of *L. innocua* is presented in Fig. 10. We suggest that the primary mechanism of TF involves ROS generation caused by the interaction between GA and H_2_O_2_ (18). As demonstrated in our previous work (21), ROS-mediated stress exerts bactericidal effects on a range of bacteria, with *Listeria* being a notable exception, perhaps due to its extraordinary defense system (10, 100). Synergistic inhibition of this bacterium was achieved after the addition of lactic acid to the GA + H_2_O_2_ combination, which likely permeabilized the cells and/or sensitized them to oxidative stress (25, 26). This synergistic TF effect inflicted sublethal injury on stress-vulnerable exponential-phase cells that might lead to death. In contrast, stationary cells, preconditioned by stress-response systems such as SigB, were able to mitigate lethality through further *sigB* upregulation. In parallel, stress-induced DNA repair through the SOS response could suppress cell division (101), leading to the VBNC phenotype (27).

The finding of this study that exponential-phase cells are less VBNC-prone than stationary-phase ones opens a possibility to improve the sanitizer efficacy by modulating the bacterial growth-phase status. The bacterial population of vegetable wash water may typically comprise non-growing stationary-phase cells transferred from the vegetable surface. Stimulating the outgrowth of these cells to exponential phase can enhance their sensitivity to antimicrobials, similar to the “germination-inactivation” strategy for controlling spore-forming bacteria (102). Rapid outgrowth of stationary-phase bacteria occurs after the supply of fresh nutrients (99) and can be encouraged by nutritional and regulatory factors (103) such as fumarate (104) or nucleoside triphosphates (105, 106). In addition, in some systems, for example, edible sprouts, the bacterial cells naturally enter exponential growth during initial sprouting stages due to highly available nutrient sources (107). Realization of this approach in real life demands further investigation, but the growing interest in practical implementation of the similar germination-inactivation principle (102) is encouraging. Understanding the genetic basis of the *Listeria*–sanitizer interaction through our ongoing transcriptomic investigation should allow the selection of additional targets for such an approach.

## Data Availability

The flow cytometry data generated in this study have been deposited in the Figshare public depository: https://figshare.com/s/f250462a9f39ce2e8389. The rest of the data generated and analyzed in this study are provided within the article and the supplementary materials.

## SUPPLEMENTAL MATERIAL

Supplemental material spectrum.03875-25-s0001.docx Fig. S1 to S6.

ASM does not own the copyrights to Supplemental Material that may be linked to, or accessed through, an article. The authors have granted ASM a non-exclusive, world-wide license to publish the Supplemental Material files. Please contact the corresponding author directly for reuse.
