# Supplementary material for: Growth phase-specific induction of a viable but nonculturable (VBNC) state in Listeria innocua in response to a green food sanitizer
Source: Microbiol Spectr. 2026 Mar 17;14(4):e03875-25. doi: 10.1128/spectrum.03875-25 (PMC13055218; doi:10.1128/spectrum.03875-25)
Supplement: Supplemental material — Fig. S1 to S6. [file spectrum.03875-25-s0001.docx]

SUPPLEMENTAL MATERIALS TO THE MANUSCRIPT

**Growth phase-specific induction of viable but nonculturable (VBNC) state**

**in *Listeria innocua* in response to a green food sanitizer**

by Esther W. Mwangi, Moshe Shemesh, Victor Rodov

Supplemental material 1.

**Performance of various organic acids as ingredients of antibacterial triple formulation (TF)**


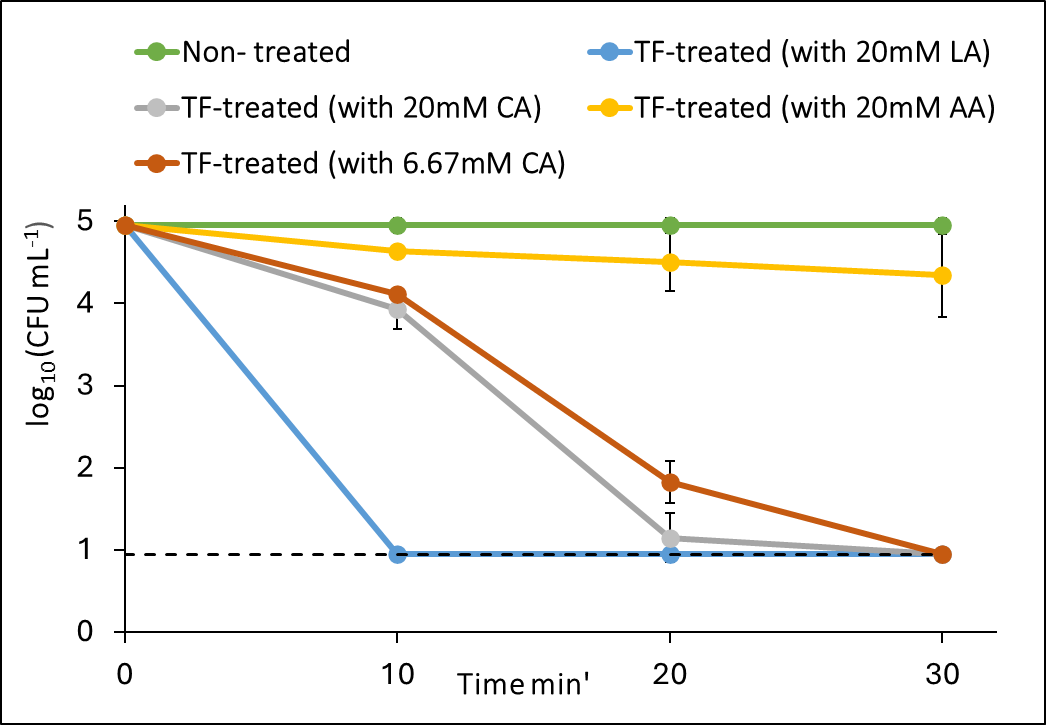


Figure S1. Culturability of stationary-phase *Listeria innocua* (5 log CFU mL^-1^) after incubation at 22 ± 1 °C in saline-based formulation comprising gallic acid (8 mM) and hydrogen peroxide (1 mM) with addition of an organic acid. Acids tested: lactic acid (LA) 20mM, acetic acid (AA) 20mM, citric acid (CA) 20mM or 6.67mM (carboxylic equivalent to 20mM LA). Non-treated control: incubation in saline without additives. Each value indicates the means of duplicate tests. Dotted black line designates the limit of detection. Error bars represent 95% confidence intervals (*p* < 0.05).

Supplemental material 2.

**Growth curve and sampling strategy of *L. innocua***

**
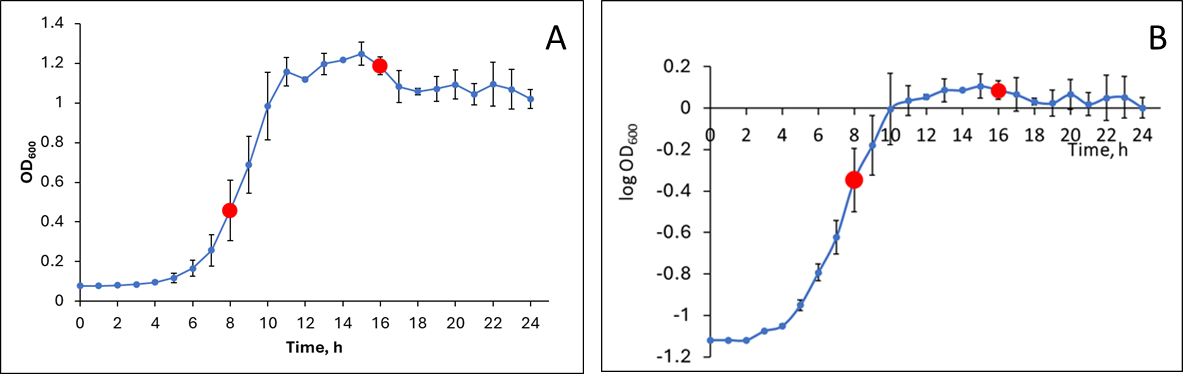
**

Figure S2. *L. innocua* growth curve in BHI broth at 37 ± 1 °C without (A) and with logarithmic transformation (B) illustrating the sampling time points (red circles) for TF treatment: exponential phase (8 h) and stationary phase (16 h). Error bars represent 95% confidence intervals (*p* < 0.05).

Supplemental material 3.

**Effect of the triple formulation (TF), its ingredients and their pairwise combinations on the culturability of low-density (5 log CFU mL^-1^) *L. innocua* cell poulations**


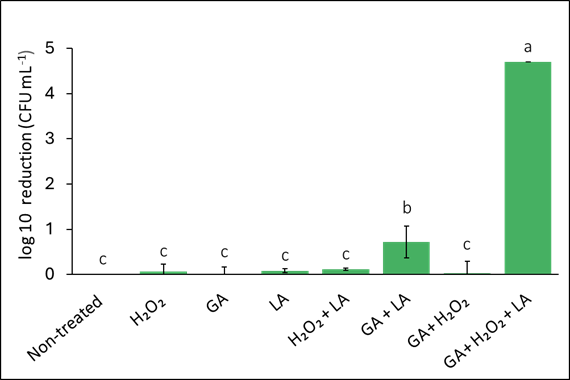


Figure S3. Culturable count reduction of *L. innocua* after 5 min incubation at 22 ± 1 °C with gallic acid (8 mM), lactic acid (20 mM), hydrogen peroxide (1 mM) and their combinations. Each value indicates the means of triplicate tests. Error bars represent 95% confidence interval. Letters indicate significant differences between treatments.

Supplemental material 4.

**VBNC induction in *L. innocua* following mild heat stress.**

**
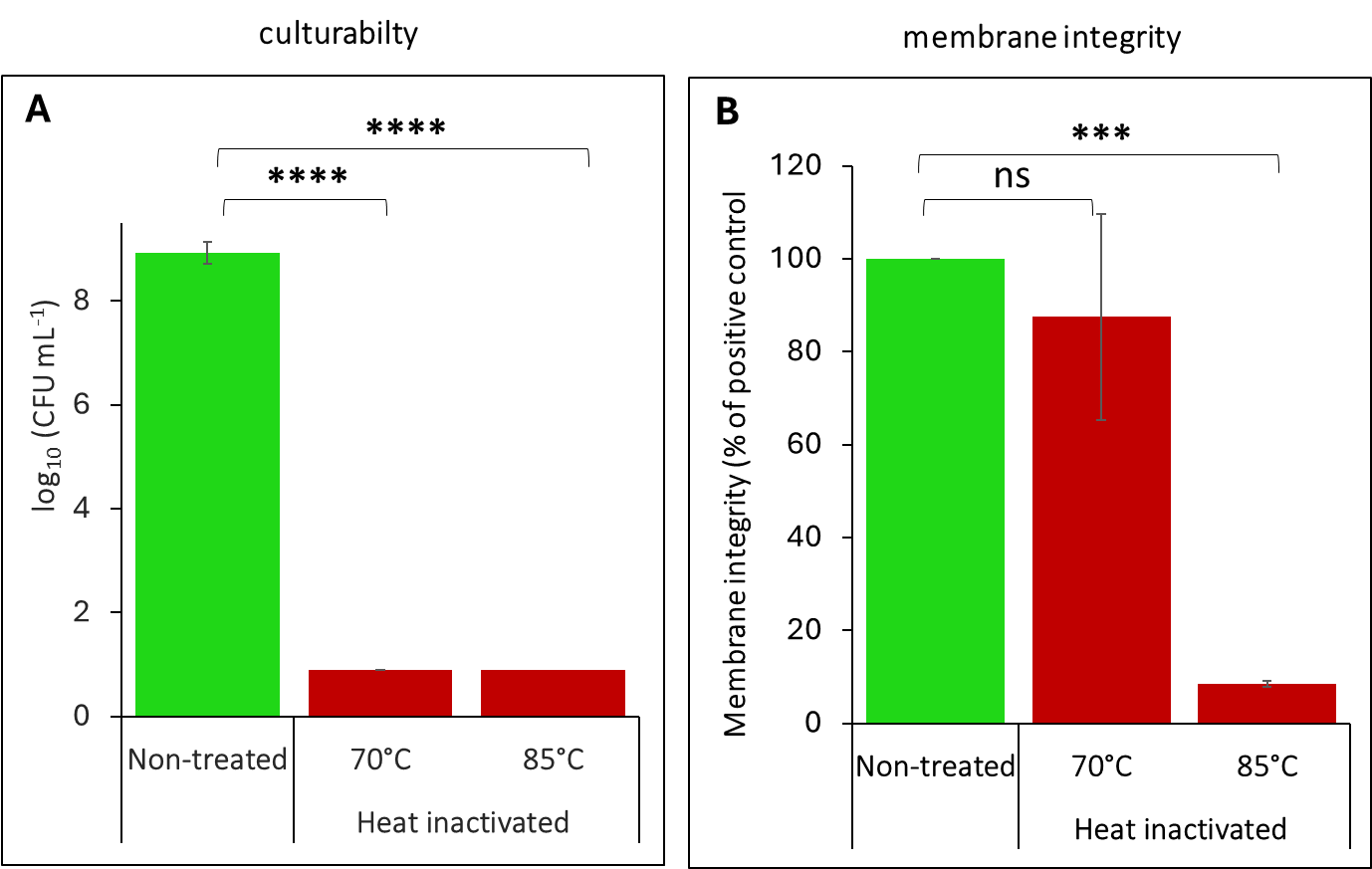
**

Figure S4. Culturability (A) and membrane integrity as determined by LIVE/DEAD™ assay (B) of *L. innocua* cells upon 10-min incubation at 70 and 85 °C. Each value indicates the means of duplicate tests. Dotted black line designates the limit of detection. Error bars represent 95% confidence intervals (*p* < 0.05). Asterisks indicate significant differences compared to control (p < 0.0001 for ****, p < 0.001 for ***, ns for non-significant).

Supplemental material 5.

**Molecular detection of VBNC in exponential- and stationary-phase *L. innocua* cells by propidium monoazide qPCR (PMAxx-vqPCR) assay**

**Methodology**

The PMAxx™ dye (Biotium, Inc., Hayward, CA, USA) staining assay was done according to manufacturer’s instructions with minor modifications. This dye is a membrane impermeable stain that prevents amplification of DNA from membrane compromised cells hence enabling selective quantification of viable cells (1). To allow dye penetration, 0.4 mL of non-treated and TF-treated suspensions were treated with 10 µM PMAxx™ and incubated in the dark for 10 minutes followed by exposure to LED (~465 nm) for 10 min in PMA-Lite; LED Photolysis Device, Biotium [E90002](https://www.ncbi.nlm.nih.gov/protein/E90002). Samples without addition of PMAxx™ were prepared in parallel. After photolysis, samples were pelleted and genomic DNA extracted using DNeasy® Powerlyzer® Microbial Kit (Qiagen, Hilden, Germany, Reference 12255-50), eluted in nuclease- free water. PCR was performed using the 16S rRNA primers and conditions similar to the ones described in the *sigB* quantification method.

Fluorescence signals were reported as cycle threshold (C_t_). The C_t_ values of PMAxx-untreated samples were subtracted from C_t_ of PMAxx-treated ones to calculate the differential dC_t_. The dC_t_ close to 0 is expected for viable cells while high dC_t_ values indicate dead cells due to PMAxx™ binding to their DNA. For instance, a 99%-inhibition of DNA amplification results in dC_t_ >4.


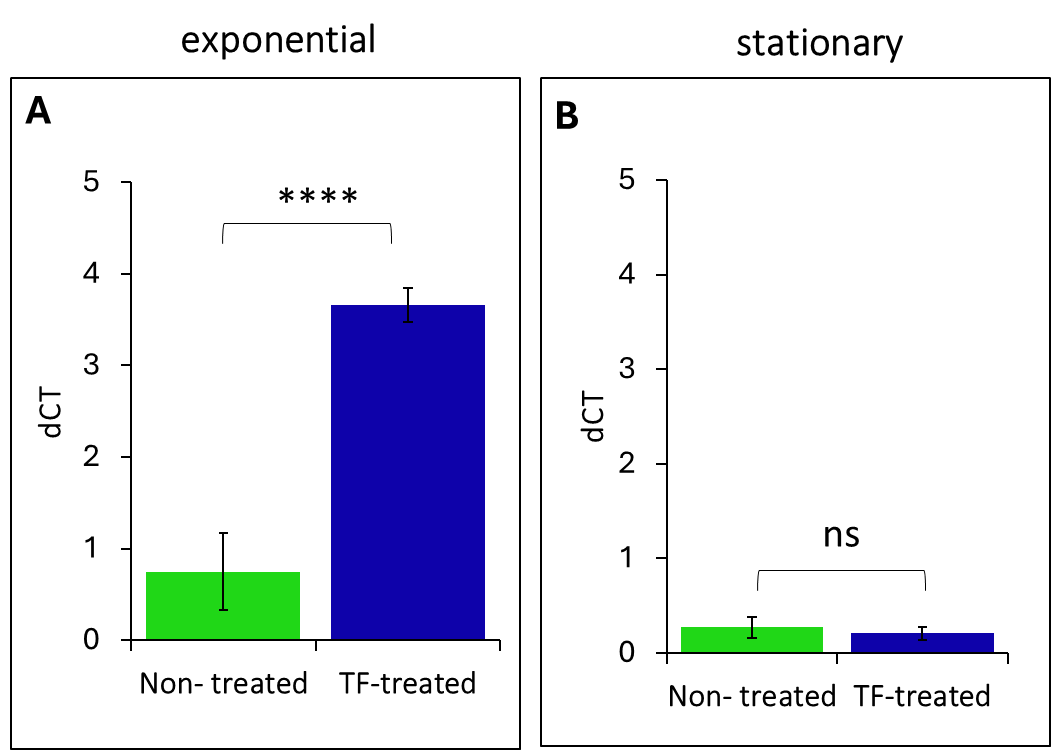


Figure S5. Difference C_t_ (dC_t_) values of 16S rRNA gene amplification in TF-treated exponential- (A) and stationary- (B) phase cells compared to non-treated controls as determined by PMAxx™ viability qPCR assay (PMAxx™ vqPCR). High dC_t_ (>1) indicates PMAxx™ dye binding meaning a compromised membrane. Data represent means of triplicate tests. Error bars represent 95% confidence interval (p < 0.05). Asterisks indicate significant differences compared to control (p < 0.0001 for ****, ns for non-significant).

Supplemental material 6.

**Resuscitation attempt of VBNC *L. innocua* using sodium pyruvate**

**Methodology**

The TF-treated, heat-inactivated (85 °C) and non-treated (control) cells were concentrated by filtration, resuspended in saline and further diluted 1:10 with BHI medium or BHI with 0.5% sodium pyruvate (SP). The cells were cultured in 96-well microplates at 37°C with growth measurement as optical density at 600 nm.


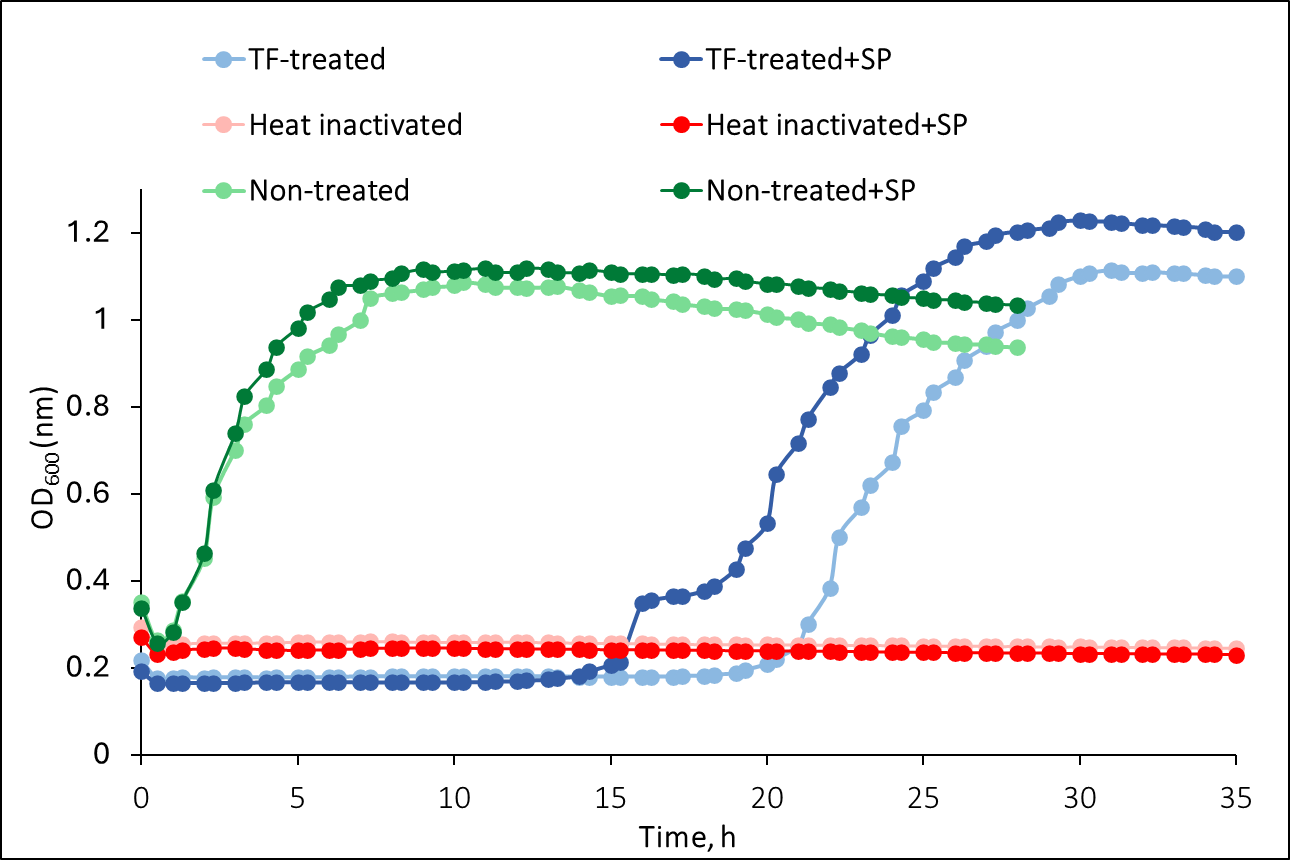


Figure S6. Effect of the resuscitation agent sodium pyruvate (SP) on the outgrowth kinetics of TF-treated, heat-inactivated and non-treated stationary-phase *L. innocua* cells. Each value indicates the means of triplicate tests.

**Results**

The non-treated control cells resumed outgrowth within 1 h after the transfer to fresh medium, while no growth was detected in heat-inactivated bacteria. The TF-treated population demonstrated regrowth after a durable 20-h lag. Sodium pyruvate shortened the lag-phase by 5 h, possibly due to a certain increase in the culturable population.
